# A multidimensional comparative study of help-seeking messages on Weibo under different stages of COVID-19 pandemic in China

**DOI:** 10.3389/fpubh.2024.1320146

**Published:** 2024-02-14

**Authors:** Jianhong Jiang, Chenyan Yao, Xinyi Song

**Affiliations:** School of Business, Guilin University of Electronic Technology, Guilin, Guangxi, China

**Keywords:** COVID-19, help-seeking behavior, social media, data mining, neural networks, regression analysis

## Abstract

**Objective:**

During the COVID-19 pandemic, people posted help-seeking messages on Weibo, a mainstream social media in China, to solve practical problems. As viruses, policies, and perceptions have all changed, help-seeking behavior on Weibo has been shown to evolve in this paper.

**Methods:**

We compare and analyze the help-seeking messages from three dimensions: content categories, time distribution, and retweeting influencing factors. First, we crawled the help-seeking messages from Weibo, and successively used CNN and xlm-roberta-large models for text classification to analyze the changes of help-seeking messages in different stages from the content categories dimension. Subsequently, we studied the time distribution of help-seeking messages and calculated the time lag using TLCC algorithm. Finally, we analyze the changes of the retweeting influencing factors of help-seeking messages in different stages by negative binomial regression.

**Results:**

(1) Help-seekers in different periods have different emphasis on content. (2) There is a significant correlation between new daily help-seeking messages and new confirmed cases in the middle stage (1/1/2022–5/20/2022), with a 16-day time lag, but there is no correlation in the latter stage (12/10/2022–2/25/2023). (3) In all the periods, pictures or videos, and the length of the text have a significant positive effect on the number of retweets of help-seeking messages, but other factors do not have exactly the same effect on the retweeting volume.

**Conclusion:**

This paper demonstrates the evolution of help-seeking messages during different stages of the COVID-19 pandemic in three dimensions: content categories, time distribution, and retweeting influencing factors, which are worthy of reference for decision-makers and help-seekers, as well as provide thinking for subsequent studies.

## 1 Introduction

The COVID-19 outbreak had become a public health emergency of international concern in recent years, causing a huge impact on people’s lives. Since the outbreak, different countries have taken their own measures to curb the spread of the virus. For China, the “Wuhan lockdown” measures implemented at the critical time in early 2020 successfully delayed the spread of the virus to other cities by an average of about 2.91 days (1). Other parts of China followed suit, and during this time, fear of the virus and the lack of medical and livelihood resources led to a severe social crisis. As a result, many people resorted to the Internet to post requests for help in order to solve real-life problems. Weibo, as one of the mainstream social media in China, plays an important role in information diffusion during the pandemic (2). According to Weibo’s Unaudited Financial Results for the Fourth Quarter and Fiscal Year 2022, published on March 1, 2023, there were 586 million monthly active Weibo users in December 2022, with a net increase of about 13 million users year-on-year. In addition, it has been demonstrated that the number of retweets of help-seeking messages on Weibo has a significant positive effect on whether help-seekers receive actual help, and the number of help-seeking messages has a positive effect on the prediction of new confirmed diagnoses (3). Therefore, the study of help-seeking messages on Weibo is of great theoretical and practical significance.

**Figure 1:**
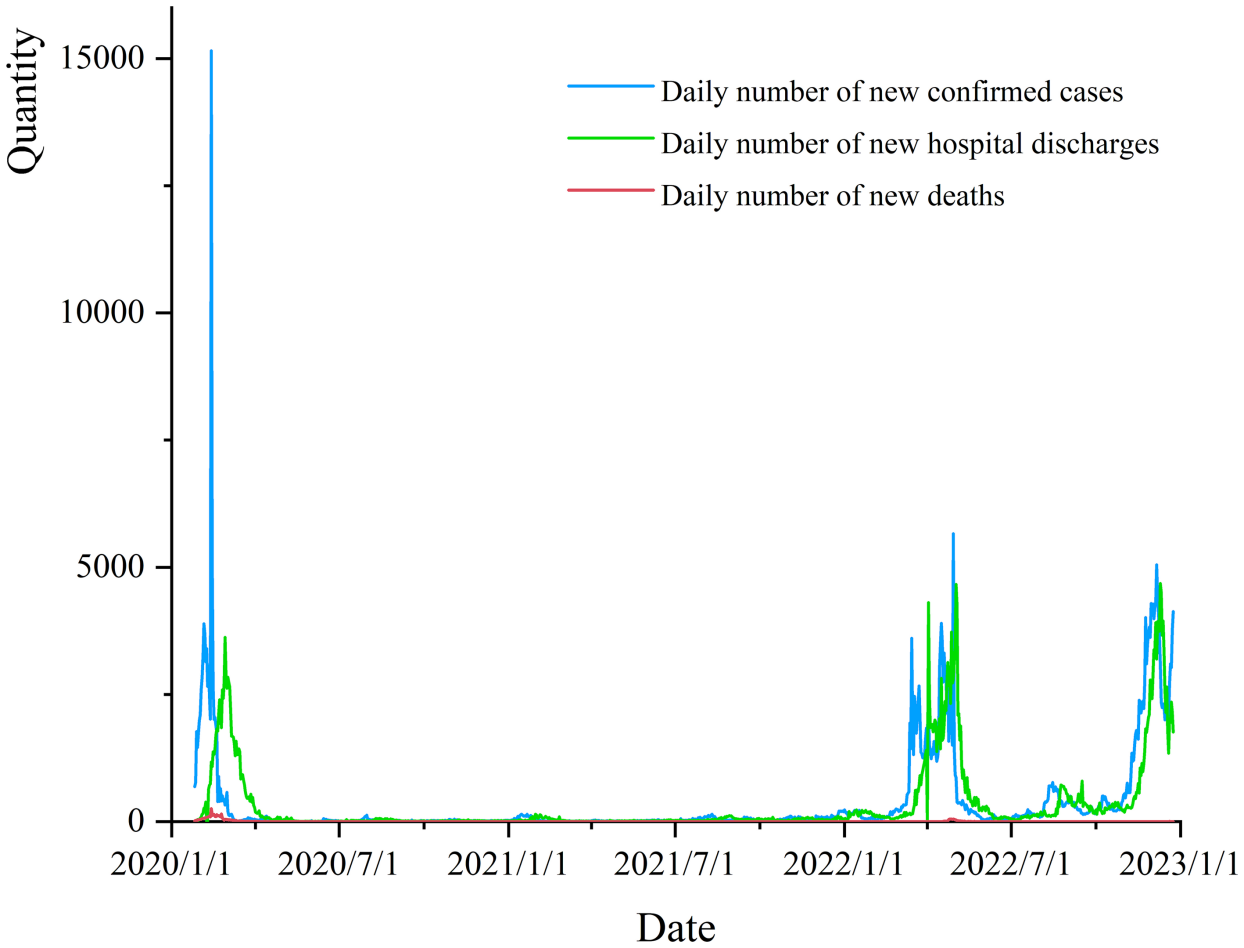
China’s new daily confirmed, discharged and dead cases.

With the duration of the pandemic increasing, viruses (4), relevant policies (5–7)and public perceptions (8–10) have changed, so how did the help-seeking messages posted on the Weibo platform change during different periods of the pandemic? This is the focus of this paper. By collecting public data from National Health Commission of China (Figure 1), the number of new daily confirmed COVID-19 cases reached its first peak in early 2020, during which the city of Wuhan was blocked in the emergency, the whole country was in a state of panic, and a large number of help-seeking messages appeared on Weibo and other social media (referred to as the **early stage** of the pandemic in the following section). The number of new daily confirmed cases reached its peak once again around March 2022, which corresponded to the periods of the Jilin and Shanghai pandemics. At this time, the national policy has been updated to “Dynamic zero-COVID,” and people have a new perception of the pandemic (referred to as the **middle stage** of the pandemic in the following section). On December 7, 2022, National Health Commission of China issued a new policy, “10 New,” and stopped the implementation of the “Dynamic zero-COVID” general policy, which means that China’s pandemic has ushered in a new stage, and the normalization of the COVID-19 pandemic has become a reality (referred to as the **later stage** of the pandemic in the following section). As a result of the policy adjustment, the number of daily new confirmed cases has reached its third peak.

This paper focuses on the help-seeking messages on Weibo in early, middle and later stages of the pandemic, and tries to investigate and empirically analyze the changes from 3 aspects: content categories, time distribution, and retweeting influencing factors. Based on the above research background, this paper proposes the following research questions:

- RQ1: Did the content of help-seeking messages on Weibo change in different periods, if so, how did the changes happen?
- RQ2: What are the changes in the temporal distribution of Weibo help-seeking messages in different periods?
- RQ3: Do the factors influencing the amount of retweets help-seeking messages can get change over time, if so, and what are the changes?

Social media is not only a tool for people to browse information and share their lives, but also plays an important role in the occurrence of public health emergencies. For example, people spread information rapidly through social media when a disaster occurs (11), and donate supplies and money to those affected by the disaster, which plays an important role in disaster relief (12). Social media is also widely used as a channel for people to seek help during public health emergencies, such as hurricanes (13), heavy rains (14–17), earthquakes (18, 19), and COVID-19 pandemic (3, 20–29).

Online help-seeking behaviors have also been proved to be related to negative emotions (30), which is a way for people to seek stress relief. In the face of public health emergencies such as earthquakes, people are not immune to showing fear and anxiety, while in the case of COVID-19 pandemic, a number of studies have demonstrated that people’s psychological stress increases during this time (31–34). When confronted with long and extensive closures such as Wuhan and Shanghai outbreaks, the reasonable scheduling of resources has also become a difficult problem faced by the administrators. During that time, some people who did not have enough medical resources or life supplies, who experienced severe anxiety, chose to post help-seeking messages on social media in order to solve the actual problems. In addition, combining with the theory of strong and weak relationships in social networks (35), relying on strong relationships such as families and friends may not be enough to support help-seekers to get help in the face of public health emergencies, thus relying on weak relationships is a wise choice. When help-seekers post request on social media, netizens generate weak relationships with help-seekers through browsing, liking, commenting, and retweeting, which provides a greater possibility for problem solving.

Scholars have already examined the help-seeking messages on Weibo (3, 20–23, 26–29), Zhihu (24), and Baidu Tieba (25) during the COVID-19 pandemic (Table 1). Until now, the COVID-19 virus has not disappeared and may even exist in people’s lives forever, but most of the current studies on help-seeking messages collected data is limited to 2020 (3, 20–29). According to Table 1, only li et al. (24) extend the timeframe (1/1/2020 – 6/30/2020) to the middle period of the pandemic, but the social platform of their study, Zhihu, is different from the Weibo in this paper. Not only that, Li et al.’s (24) research still does not cover the later stage of the pandemic. For China, in addition to the outbreak period represented by the closure of Wuhan at the beginning of 2020, the Jilin and Shanghai outbreaks in 2022 and the implementation of the open-door policy at the end of 2022 both led to peaks of the pandemic. During the latter two peak outbreaks, there are also a large number of help-seeking messages on the internet, which in themselves are of significant research value, but have not been adequately studied. This paper not only complements the research on the latter two periods, but also systematically compares how help-seeking messages have changed over time in three dimensions that no research has ever done before. As for the choice of social platforms, Weibo is one of the traditional and mainstream social media in China, similar to Twitter and Facebook, where users can share their lives and express various emotions. Compared to Baidu Tieba or Zhihu, Weibo has more active users and great interactivity, which means the help-seeking messages are likely to get more readers.

**Table 1:** Studies on help-seeking messages during the COVID-19 pandemic.

Authors	Social media	Period of the COVID-19 pandemic	Objective	Methods	Findings
Zhou et al. (3)	Weibo	Early period	Exploring whether and to what extent the help-seeking crying could be heard at the individual level	Granger causality, bert for text classification, textual analysis, logistic regression	Help-seeking message had a Granger causality with the number of new diagnose, with an 8-day time lag. Constructing the content-context-connection(3C) framework. The amounts of retweets had a significant effect on whether or not actual help was received, while comments did not.
Luo et al. (20)	Weibo	Early period	Exploring the factors that influence the dissemination of help-seeking messages	Negative binomial regression, textual analysis	Posts release anger, express instrumental support seeking intention, report self-illness, expound suspected cases’ conditions, and have detailed individual information disclosure were found to be more likely to gain retweets.
Yang et al. (21)	Weibo	Early period	Exploring the role that Weibo plays when users are seeking help, as well as exploring the room for improvement in Weibo	Mixed-methods analysis combining bert, controlled interrupted time series analysis, regression analysis, etc.	Existing Weibo functionality needs to be improved in several ways, including capabilities of search and tracking requests, ease of use, and privacy protection.
Guo et al. (22)	Weibo	Early period	Exploring the textual features of Weibo help-seeking messages during the first closure of Wuhan and the impact of these textual features on user engagement	Textual analysis, sentiment analysis, negative binomial regression	Summarizing the type of help-seeking messages, the narrative, the publisher, and the sentiment, and further exploring the impact of these four aspects on retweets and comments.
Chen et al. (23)	Weibo	Early period	Exploring the factors influencing the depth of diffusion of help-seeking messages	Textual analysis, sentiment analysis, negative binomial regression	Sender, post content, and situational factors can impact the diffusion depth of messages.
Li et al. (24)	Zhihu	Early period and middle period	Exploring the relationship between the number of new diagnoses and the number of help-seeking messages	LDA model, textual analysis, sentiment analysis	The number of new diagnoses and the number of help-seeking messages were shown to be significantly positively correlated. People were most concerned about quarantine assistance and quarantine locations, and that the public was more negatively disposed.
Liu et al. (25)	Baidu Tieba	Early period	Exploring the support-seeking strategies and social support offered on the online forum “Baidu COVID-19 bar”	Textual analysis	Users’ main help-seeking measures were asking for support and disclosing directly, while the former was more likely to elicit informational support and the latter was more likely to elicit emotional support.
Sun et al. (26)	Weibo	Early period	Exploring Weibo users’ responses to help-seekers looking for support	Textual analysis	The content characteristics of help-seeking message were found to influence retweets, comments, and likes. The type of support fell into three categories: emotional, informational, and diffusional supports.
Chen et al. (27)	Weibo	Early period	Exploring the motivations and strategies of commenters of help-seeking messages	Interview	Interviews with 23 users in the Weibo Super Topic revealed that geographic proximity and level of expertise influenced users’ commenting behavior.
Huang et al. (28)	Weibo	Early period	Analyzing the characteristics of the Weibo help-seekers’ medical conditions	Textual analysis	Most of the help-seekers were elderly people living in Wuhan, most of them had febrile symptoms, and ground-glass opacities were noted in chest computed tomography. Family aggregation of infections was easy to occur.
Zhao et al. (29)	Weibo	Early period	Exploring how help-seekers use the Internet to seek health information	Entity identification, textual analysis	Chinese citizens use the Internet as an important source of health information. The most searched information included accessing medical treatment, managing self-quarantine, and offline to online support.

In addition, this paper is not limited to only studying the phenomenon, but also seeks to draw valuable meanings for reality from the phenomenon. For example, the content and quantity of help-seeking messages reflect the current social problems and public needs to a certain extent, the significant correlation between the number of new diagnose and the number of help-seeking messages also represents whether the help-seeking messages can predict the number of new diagnose, and the forwarding influence factor of help-seeking messages relates to the exposure degree of the help-seeking messages posted by the help-seekers, which also affects whether they get actual help to a certain extent. While examining these three dimensions of help-seeking messages, this paper summarizes recommendations for policy makers and help-seekers, which are contributions that are unique to this paper and not available in the existing research.

Previous studies have found that help-seeking messages face the risk of being flooded by other information containing the same keywords (17). How to improve the effectiveness of help-seeking messages diffusion without drowning it in irrelevant messages is an issue that all help-seekers must consider, and it is also one of the focuses of scholars’ research.

**Figure 2:**
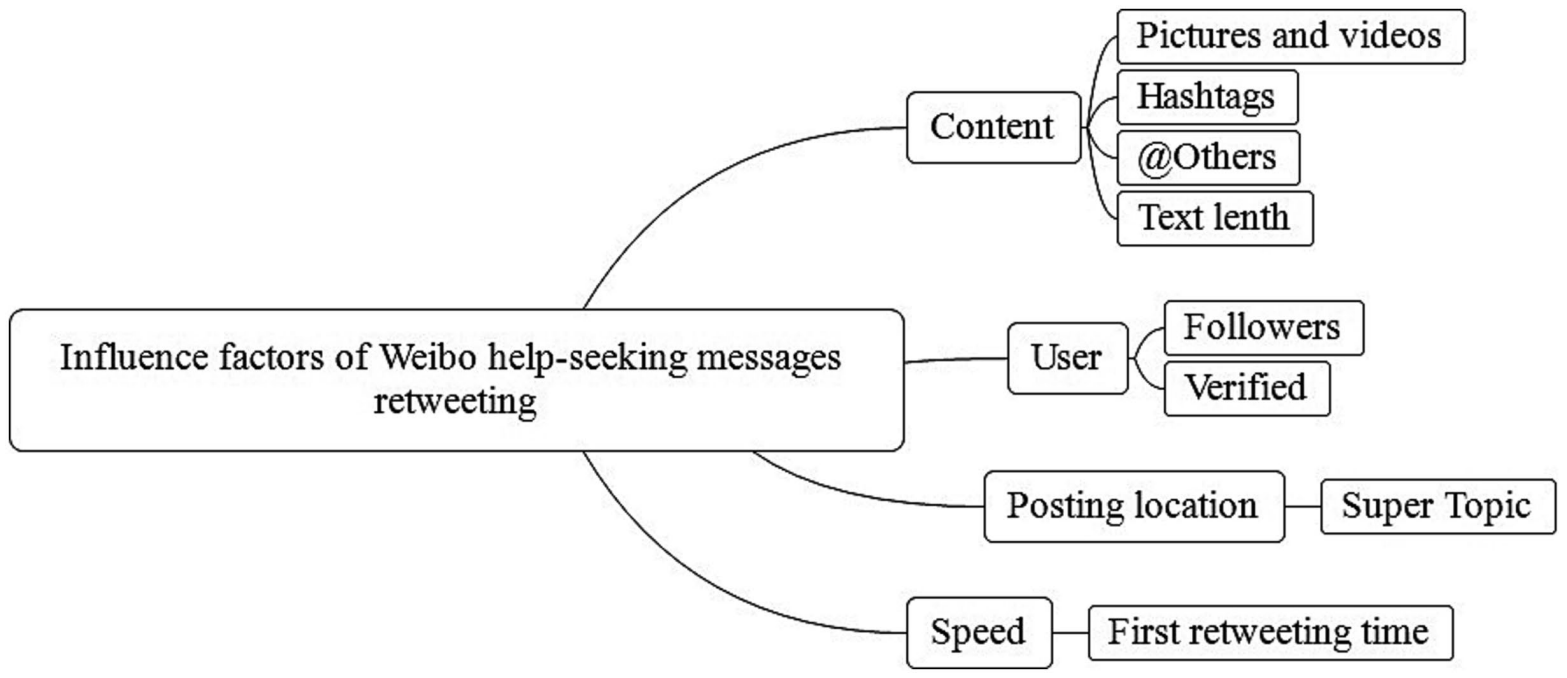
The model of influencing factors for Weibo help-seeking messages retweeting.

Existing research on information diffusion mainly focuses on diffusion size (36), breadth, depth (37), and speed (38). Narrowing the context to the Weibo during the COVID-19 pandemic, the diffusion size is usually measured by the number of retweets or comments. The diffusion breadth usually refers to the total number of first-level sub-nodes, and the depth of diffusion refers to the length of the diffusion path of the help-seeking information on Weibo. The speed refers to the efficiency of the information diffusion process. Scholars have researched the influencing factors of Weibo help-seeking messages on Weibo in the early pandemic from different aspects such as diffusion size (3, 20, 27) and diffusion depth (23). In terms of the selection of factors, researchers generally agree that content characteristics and posting user’s characteristics can significantly contribute to the diffusion of the message (21, 39, 40). For example, Suh et al. (41) collected 74 million posts on Twitter and found that in terms of content, the inclusion of URLs and hashtags had significant effects on the number of retweets. In terms of users, the number of followers and followings, and the age of the user’s account also had significant effects on getting retweets. The location of the message is also important (42). Zhou et al. (3) proposed the content-context-connection(3C) framework to explain the effect of message dissemination on social media, and the location of the message, such as posting in hypertext or posting in Hubei, is included in the “context.” Besides the three factors of content, user and posting location, this paper will innovatively examine the effect of how quickly a message is first reposted on the size of the diffusion (Figure 2).

There is a relative abundance of research on the factors influencing the retweeting of help-seeking messages in the early period of the pandemic (3, 20, 23, 27), but there is a lack of empirical studies on this issue for the middle and later stages of the pandemic. This paper complements existing research on the factors influencing the retweeting of help-seeking information in other periods by exploring stable factors that have an impact on retweets’ number in all periods, as well as unstable factors that only play a significant role in individual periods, so as to provide advice and assistance to help-seekers (RQ3).

Currently, there is a relative abundance of research on the influence of content factors of messages on retweets, while each article varies in the choice of factors for content features. In this paper, we combine the results of existing studies and the characteristics of the current data to select six factors of content features, which are pictures or videos, hashtags, @others, text length, sentiment intensity, and content categories.

The completeness of information has been shown to increase credibility, which has a positive effect on the diffusion of information (43). Adding pictures or videos (20) will make the message richer and easier to understand than the content of text alone. With the corroboration of the pictures or videos, the help-seeking messages itself also gains higher credibility, which in turn brings more retweets to the messages (44, 45). Furthermore, the use of “#” in messages can quickly categorize different types of information, and it also facilitates users to search for a specific topic. When disaster strikes, the help-seekers seem to subconsciously use “#” as a way to increase the effectiveness of information dissemination. Specifically, during the flooding in Houston, help-seekers used hashtags such as “#HarveySOS” in order to gain more attention (46), and the same happened in the early stage of the COVID-19 pandemic (29). The use of “#” has also been repeatedly proved to have a positive effect on the diffusion of information (17, 41, 47). Other than “#,” messages can also use the symbol “@” to enhance the directional function of information transmission. When disaster strikes, it is wise to @ the more influential people. Numerous studies have demonstrated the positive effect of @ on information diffusion (21, 48, 49), and Zhou et al. (3) also confirmed the positive effect of @ on the number of retweets in their study of help-seeking messages in the early stage of the COVID-19 pandemic.

Furthermore, text length is often considered to be related to message completeness. For help-seeking messages, having a larger word count may mean that this message contains more information, such as condition, address, phone number, etc. (17), and may likewise have a richer sentiment, which can have a positive impact on the diffusion of the message (47). Research has shown that text length is an important indicator of increased credibility (50) and also has a significant positive effect on Weibo retweets (16). Emotion types are generally categorized as positive, negative, and neutral emotions. During the COVID-19 pandemic, most of the help-seeking messages in social media contained negative emotions. Messages with emotional words tend to attract more attention from other users than narratives with a calm tone (51), thus getting more retweets (52–54). In contrast, messages with negative emotions tend to be more likely to be spread compared to positive emotions (54). Among negative emotions, anger and anxiety are more likely to stimulate psychological responses in readers, thus promoting higher emotional identification, which in turn causes retweeting behavior. Finally, in terms of the effect of content categories on the amount of retweets, it has been confirmed in previous research that different topics have different effects on the retweets of messages (3). Users will produce different behavioral choices for different types of themes of Weibo, for example, the message of strong request for hospitalization tends to have a more emotional expression compared to the information in the category of advice, and readers can clearly distinguish the degree of urgency of each when receiving both messages, which in turn affects the forwarding behavior. Therefore, the effect of content category on the retweets of help-seeking messages should not be ignored.

Synthesizing the above studies, we propose the following hypotheses:

Help-seeking messages containing pictures or videos will receive more retweets in the middle (*H1*a) or later (*H1*b) pandemic period.

Help-seeking messages containing hashtags will receive more retweets in the middle (*H2*a) or later stage (*H2*b) of the pandemic.

Help-seeking messages containing “@” will receive more retweets in the middle (*H3*a) or later(*H3*b) pandemic period.

Help-seeking messages with longer text lengths will receive more retweets in the middle (*H4*a) or later (*H4*b) period of the pandemic.

Help-seeking messages with stronger negative sentiment intensity will receive more retweets in the middle (*H5*a) or later (*H5*b) period of the pandemic.

The content categories of help-seeking messages in the middle (*H6*a) or later (*H6*b) pandemic period affect the number of retweets.

User characteristics can be even more important than message content characteristics in the degree of influence on message retweeting, such as the number of followers a user has (41) and being verified or not (3). The number of followers implies the influence of the user, which means that the messages are more likely to be seen by more people, and thus more likely to be retweeted. Suh et al. (41) found that the number of followers of a twitter user has a significant positive effect on the number of retweets, which was also verified by Petrovic et al. (49). Whether a user is verified or not is another indicator of a user’s influence, as verified users tend to be more authoritative than other users, which gives them an advantage in information diffusion. It has been shown that whether a user is verified or not has a significant positive effect on the amount of forwarding of help messages during the COVID-19 pandemic (3). Therefore, based on the above research, the following hypotheses are proposed in this paper:

Help-seeking messages posted by users with more followers receive more retweets in the middle (*H7*a) or late (*H7*b) period of the pandemic.

The verified users who post help-seeking messages in the middle (*H8*a) or later (*H8*b) period of the pandemic will receive more retweets.

“Super Topic” is a community based on common interests, topics and interactive features integrated in a single place in Sina Weibo, where one can get more focused access to relevant information under the same topic. During the COVID-19 pandemic, “Super Topic” played the role of a mutual support community and created a pure, non-intrusive, and mutually helpful atmosphere, where irrelevant information was reviewed by administrators, and volunteers monitored the help-seeking posts in “Super Topic” on a daily basis and coordinated with the relevant departments (55), which facilitated the resolution of the task from online help-seeking to offline treatment. In addition to the early construction of the “COVID-19 patient help Super Topic,” there have been “Jilin help against pandemic Super Topic,” “Shanghai help against pandemic Super Topic,” and finally merged into the “Help against pandemic Super Topic.” The total number of followers of all the “Super Topic” exceeded 500,000. In summary, this paper concludes that “Super Topic” plays a positive role in the diffusion of help-seeking messages:

In the middle (*H9*a) or later (*H9*b) period of the COVID-19 pandemic, messages posted in “Super Topic” had a positive impact on the number of retweets.

The concept of speed was first proposed by Yang and Counts (38), where speed is whether and when the first forwarding occurs. In previous studies, the speed and the size of information diffusion have been considered as different evaluation indexes, and the effect of speed on the amount of retweets has rarely been studied. There is no empirical study to prove whether the speed of the first forwarding time has an effect on the total number of retweets in the case of the diffusion of help-seeking messages. Therefore, it is of sufficient theoretical and practical significance to prove the influence of the speed dimension on the total number of retweets of help-seeking information. This paper makes the following hypotheses:

The shorter the time of the first time being retweeted, the more retweets the posts have for the help-seeking messages in the middle (*H1*0a) or later (*H1*0b) period of the pandemic.

## 2 Methods

### 2.1 Data collection and processing

For the outbreak of the Jilin pandemic and Shanghai pandemic around March 2022, we crawled the posts on Weibo twice in March and May 2022 using Python, and collected all original messages containing the keywords (“*Jilin help*,” “*Changchun help*,” “*pandemic help*,” “*help against pandemic*”, “*Shanghai help*”,) on Weibo from January 1, 2022 to May 20, 2022 (*N* = 87,314, *N* refers the total number of messages in our dataset). Then we filter out the messages with wrong format, resulting a dataset with *N* = 87,244.

The next step is to select all the help-seeking messages and eliminate irrelevant ones. We model this process as a binary topic classification problem. Specifically, we train a classifier that takes a piece of message as input, and determines if that message is related to help-seeking or not. In order to train this model, we randomly sampled 2,400 posts, and manually labeled whether they were related to help-seeking or not. Then, we use 2,000 posts as training data and 400 posts as test data to train the topic classifier, of which half of the training data are for help-seeking or not. We experimented with both Convolutional Neural Network (CNN) (56, 57) and Recurrent Neural Network (RNN) (58) for our topic classification model. The most straightforward setting is to use the content of the messages as the input of the neural network. However, where the messages were posted (e.g., under the *help against pandemic Super Topic*) might also be helpful for the classifier. Therefore, we also experimented with combining the content and the “Publishing Tool” of a message as the input of our CNN or RNN models. We show the results of the 4 settings in Table 2.

**Table 2:** Comparison of topic classification models evaluation metrics.

Models name	Accuracy	precision	Recall	F1
CNN—“text of posts”	0.935	0.935	0.935	0.935
RNN—“text of posts”	0.925	0.925	0.925	0.925
**CNN—“text of posts” combined with “publishing tool”**	**0.948**	**0.950**	**0.948**	**0.947**
RNN—“text of posts” combined with “publishing tool”	0.938	0.938	0.938	0.937

As showed in Table 2, our RNN models have lower performance than the CNN models. The CNN based model achieves 0.935 F1 score with only the “text of posts” as input. Adding “Publishing Location” as part of the input further increases the F1 score of the CNN model to 0.947. We take our best performing model, CNN—“text of posts” combined with “publishing tool,” and conduct classification on the rest of the data. As a result, 17,834 help-seeking messages are obtained.

The next step is to crawl the user information and retweeting information of the help-seeking messages. There was a time gap between our previous Weibo crawling practice and this step, we found that there were users who deleted some of their posts or made them private, there were also users who deleted their Weibo accounts. Due to those reasons, we were not able to get the user and retweeting information of all the messages we have crawled. For the sake of data rigor, we decided to only keep the data that we could get all the information. As a result, we have 15,197 messages with user information and retweeting data in our dataset.

For the Weibo help-seeking information at the later stage of the COVID-19 pandemic, we crawled all the original Weibo messages from December 10, 2022 to February 25, 2023 containing the keywords “*help against pandemic*,” “*COVID-19 help*” or “*white lung help*” in February 2023 (*N* = 5,891). We manually selected 830 help messages and crawled all their retweets and user information.

In the end, we obtained an analyzed a corpus of 16,027 original help-seeking posts in the middle and later stages of the pandemic, as well as all their retweets and users information.

### 2.2 Topic classification

After looking into the data carefully, we classify the help-seeking messages in our dataset into different categories based on their topics. However, we did not use the same set of categories for both the middle and later stage of the pandemic, as the content and focus of the help-seeking messages of those two stages were different. For example, messages in the later period were no longer focused on “other diseases patients seeking drugs” or “seeking supplies,” while the proportion of “COVID-19 patients seeking drugs” increased significantly. Furthermore, a new type of help-seeking information emerged in the later period, namely, “selling drugs.” Due to the liberalization of the policy and the widespread outbreak of pandemics, coupled with the fact that some people hoarded large quantities of medicines out of a sense of security or other psychological reasons, medical resources were insufficient to cope with the nation’s needs, and thus information about seeking medicines appeared on the Internet. At the same time, those who have surplus medicines wish to pass the medicines to others, whether they do so out of goodwill or just for the sake of gaining profit. More details about those categories are showed in Table 3.

**Table 3:** Content categories of help-seeking messages.

Periods	Categories	Description
Middle period (1/1/2022 – 5/20/2022)	COVID-19 patients seeking hospital treatment	COVID-19 patient with or without underlying disease seeks help for hospital treatment.
	COVID-19 patients seeking drugs	COVID-19 patients with or without underlying disease seek drugs.
	Other diseases patients seeking hospital treatment	Patients with other diseases who are not infected with the COVID-19 virus seek hospital treatment.
	Other diseases patients seeking drugs	Patients with other diseases who are cut off from drugs simply seeking drugs.
	Seeking supplies	Lack of foods, immunization items, and other supplies.
	Seeking help to optimize management measures	Seeking help because of problems caused by inappropriate management measures, such as quarantine, containment, transportation, or inappropriate management of neighborhoods or streets.
	Problem counseling	Those who are confused about a problem and ask for a solution on Weibo.
	Other	Those who do not fall into the above categories are categorized as other.
Later period (12/10/2022 – 2/25/2023)	COVID-19 patients seeking hospital treatment	COVID-19 patient with or without underlying disease seeks help for hospital treatment.
	COVID-19 patients seeking drugs	COVID-19 patients with or without underlying disease seek drugs.
	Other diseases patients seeking hospital treatment	Patients with other diseases who are cut off from drugs simply seeking drugs.
	Selling drugs	Wish to pass on surplus or hoarded drugs to others.
	Seeking help to optimize management measures	Seeking help because of life distress caused by inappropriate management practices, such as liberalized policies and hospital mismanagement.
	Problem counseling	Those who are confused about a problem and ask for a solution on Weibo.
	Other	Those who do not fall into the above categories are categorized as other.

For middle stage data, In order to train a classifier for topic classification, we randomly sampled 2,230 instances as the training set, 195 instances as the validation set, and 223 instances as the test set, and manually annotate those instances to get gold labels. Then, we finetuned 3 pre-trained language models, bert-base-chinese (59), xlm-roberta-base (60) and xlm-roberta-large (60), with the input of the models as “text of posts” and the output being one of the 8 categories. As showed in Table 4, xlm-roberta-large model outperforms both bert-base-chinese and xlm-roberta-base models, achieves the best results under all metrics. Therefore, xlm-roberta-large is chosen as the model for topic classification in this period.

**Table 4:** Classification models metrics in different datasets.

Data	Models name	Accuracy	precision	Recall	F1
Test	bert-base-chinese	0.816	0.826	0.816	0.814
	xlm-roberta-base	0.794	0.805	0.794	0.792
	**xlm-roberta-large**	**0.825**	**0.832**	**0.825**	**0.823**
Validation	bert-base-chinese	0.831	0.839	0.831	0.830
	xlm-roberta-base	0.779	0.787	0.779	0.772
	**xlm-roberta-large**	**0.836**	**0.841**	**0.836**	**0.834**

Since the later period and middle period data show different emphases, and the amount of later period data was greatly reduced compared with the middle period, we manually classified the late period data into 7 categories (Table 3).

### 2.3 Retweeting influencing factors

#### 2.3.1 Emotional intensity

Since most of the current research on textual emotions focuses on distinguishing emotional polarity, i.e., doing the categorization of positive, negative, and neutral emotions, it does not appropriately express the emotional intensity of the text, especially the online help-seeking messages in the context of pandemic. The data in this paper is characterized by the fact that the sentiment polarity of the help-seeking messages during the disaster period is at most neutral, and the rest are all negative sentiment messages. How to make further distinction for these negative sentiment messages is the focus of this section. Existing common tools for detecting affective strength include SentiStrength, but due to its language limitation and the fact that the score is limited to only 5 to −5 points, which is not applicable to this data (61).

In this paper, we choose sentiment dictionary to calculate the sentiment strength by counting the number of occurrences of positive or negative words, and finally summarizing the scores to get the sentiment strength value. Specifically, this paper chooses the Hownet sentiment dictionary to assist in completing this work. This dictionary is widely used in academic research (62), and compared with other Chinese dictionaries, it’s advantage is that it comes with dictionaries of adverbs of degree, such as the level of “most,” “very,” “more” and “ish,” etc. In addition, the Hownet sentiment dictionary also comes with negative and positive sentiment dictionaries. Considering that strong words increase the intensity of emotions, this paper assigns weights to the “most,” “very,” “more” and “ish” dictionaries, which are 4, 3, 2 and 1.5, respectively. When a help-seeking message enters the program, its computational flow formula is shown below:


$$y_{r}=\overset{n_{r}}{\underset{m=1}{\sum }}\left(X_{m}-x_{m}\right)\left(1+4a_{m}\right)\left(1+3b_{m}\right)\left(1+2c_{m}\right)\left(1+1.5d_{m}\right)$$

*y*_r_ denotes the sentiment intensity of the *r*-th help-seeking message. The *r*-th help message is divided into nr phrases by the Jieba library before it is retrieved by the dictionary, and the number of phrases in each help-seeking message depends on the message itself. Based on the Hownet sentiment dictionary, the program retrieves out the number of positive sentiment words (*X*_m_), the number of negative sentiment words (*x*_m_), and the number of adverbs of different degrees in each phrase, and then multiply them by the formula and add them up to get the value of the sentiment intensity of each help-seeking message.

### 2.4 Speed

In this paper, the first forwarding time is used as a proxy for speed. It is calculated as the difference between the posting time and the first forwarding time of a request for help-seeking message, and converted to a numerical format in minutes, e.g., 30 s is recorded as the number 0.5, 1 h is recorded as the number 60, and so on. Considering that some help-seeking messages are not forwarded, in this paper, the first forwarding time corresponding to these help-seeking messages is recorded as the maximum value of 1,000,000.

## 3 Results

### 3.1 RQ1: the content difference of help-seeking messages in different stages of the COVID-19 pandemic

**Figure 3:**
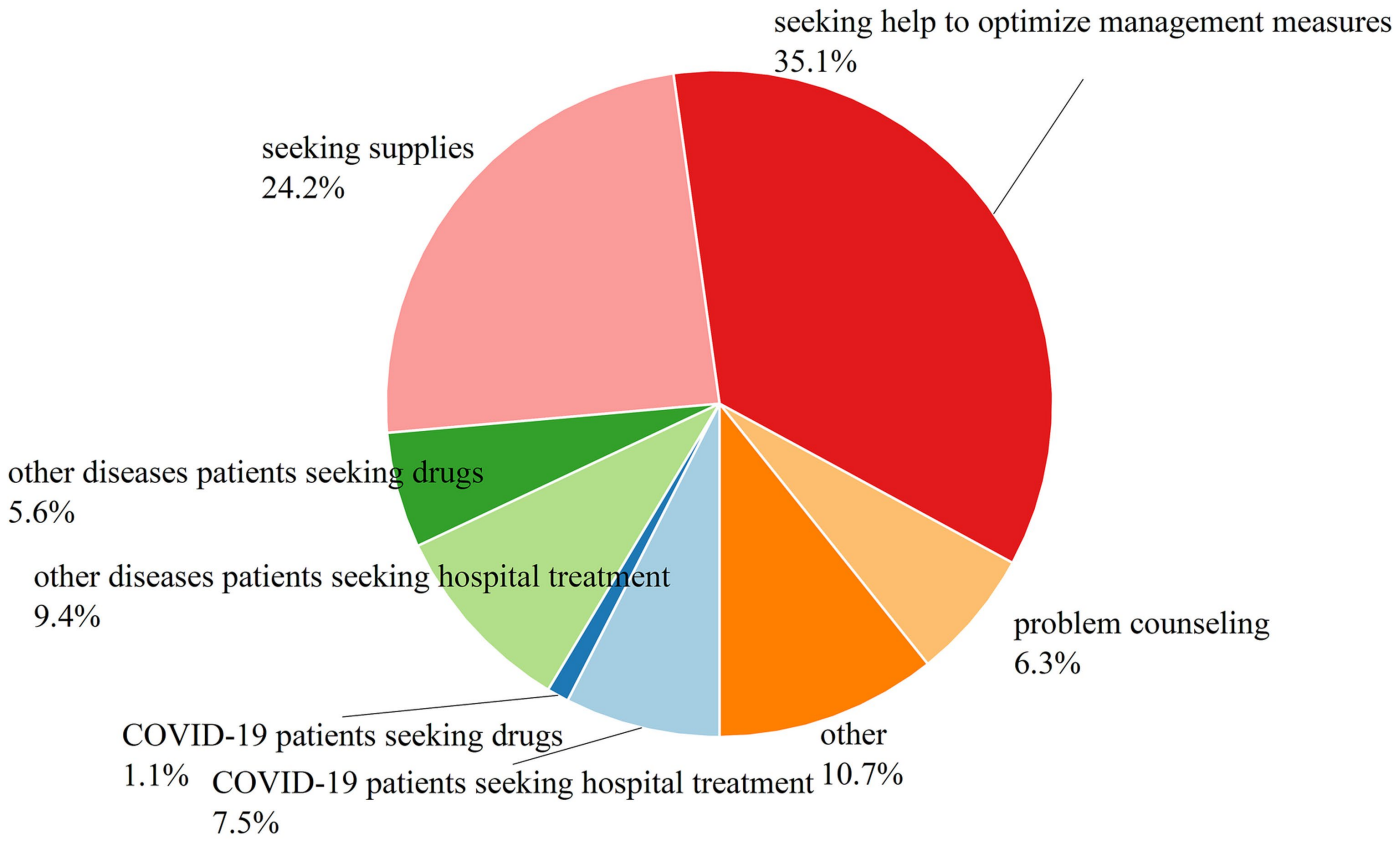
Results of the content classification of help-seeking messages in the middle period.

According to Figure 3, among the categories of help-seeking messages in the middle stage of the pandemic, the category of “seeking help to optimize management measures” accounted for the largest share of 35%, in which help-seekers were mostly troubled by problems such as “untimely transfer of positive patients from the same community,” “poor quarantine environment,” “disinfection not in place” and “inaction of the neighborhood committee,” which reminds managers that these problems are likely to occur during the centralized closure and control period, and to make corrections. The “seeking supplies” category accounted for the second share of 24%. Under the “Dynamic COVID-zero” policy, the pandemic has been effectively controlled, however, it is inevitable that residents who were sealed off and asked to stay at home would not have enough supplies. Therefore, a large number of requests for supplies information appeared on the Internet at this stage.

Compared with “COVID-19 patients seeking hospital treatment,” more instances fall into the category of “other diseases patients seeking hospital treatment.” When patients with other diseases looking for hospital treatment, most of them need chemotherapy or blood dialysis, and most of them are not infected with the COVID-19 virus. The reasons that those patients do not have access to hospitalization are because of the lack of space and resources in hospitals, and the difficulty of transportation caused by lockdown, etc. This shows that the pandemic closure also affects the treatment of other diseases. The reason for the lower number of “COVID-19 patients seeking hospital treatment” is that during the middle pandemic period in Shanghai, the Square Cabin Hospital and the sentinel hospitals were well established, and there were a set of separate procedures for COVID-19 patients seeking hospital treatment. The category of “other diseases patients seeking drugs” accounted for 6% of the total, indicating that the pandemic closure and control also caused problems for people who had been taking therapeutic drugs for other diseases for a long time. During this period, very few help-seekers posted information that was solely for the drugs used to treat the COVID-19, and the proportion of “COVID-19 patients seeking drugs” was only 1%. Otherwise, the share of “other” category is 11%, and the share of “problem counseling” category is 7%.

**Figure 4:**
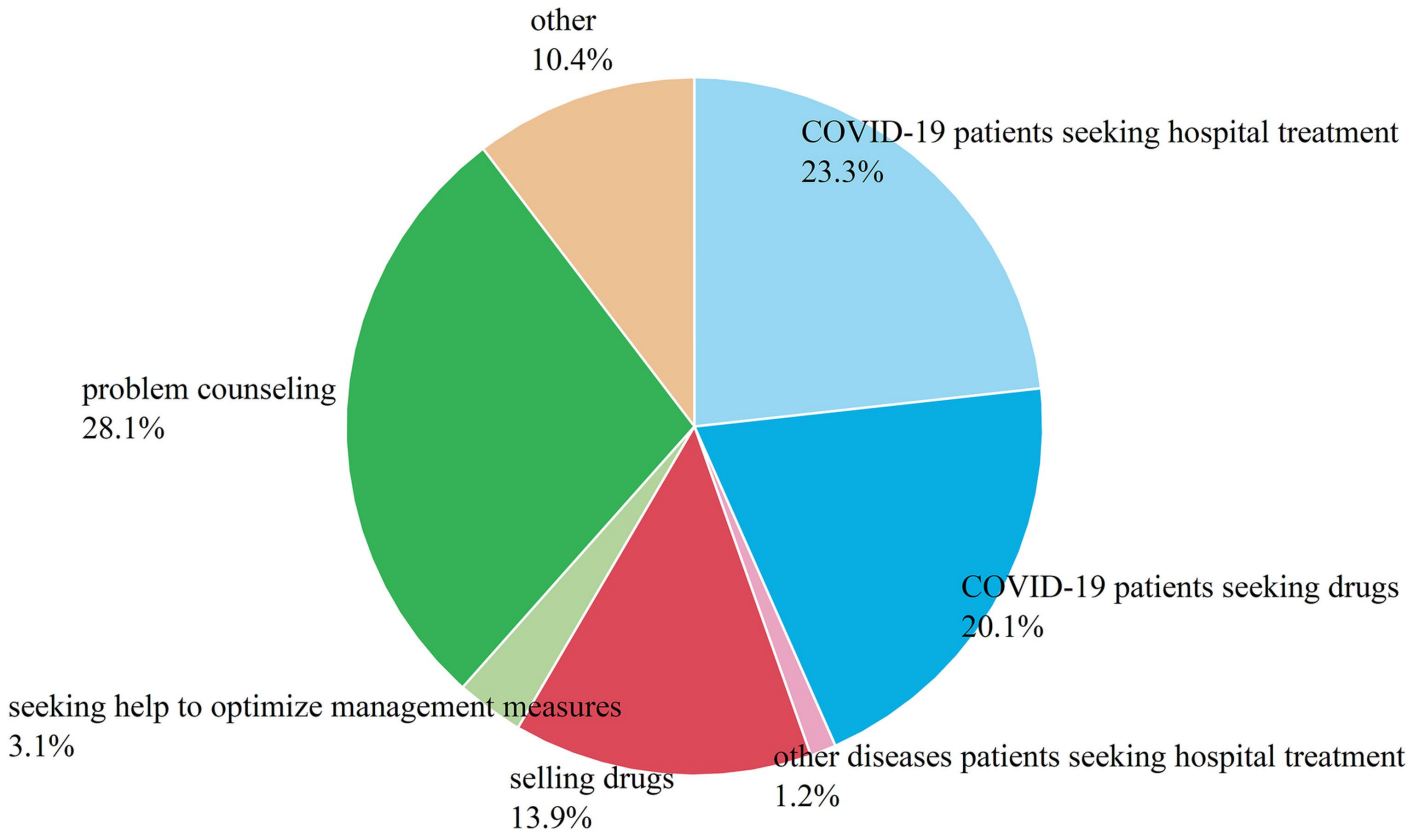
Results of the content classification of help-seeking messages in the later period.

According to Figure 4, among the categories of help-seeking information in the later stage of the COVID-19 pandemic, the category of “problem consulting” accounted for the first share, as high as 28%, which may be due to the fact that after the liberalization of the pandemic, most of the COVID-19 patients, except for those with severe illnesses, chose to prepare their own medicines for treatment. For the general public who lacked the relevant medical knowledge, posting consulting questions on social media would give them a chance to hear the opinions of medical professionals or people with more experiences, thus achieving the purpose of consulting. The posters of “COVID-19 patients seeking hospital treatment” and “COVID-19 patients seeking drugs” are mostly family members of severely COVID-19 patients, with the keywords “white lungs” and “blood oxygen” attracting more attention, which also reminds the public of the need to enhance their awareness of the risks of COVID-19. The category of “selling drugs” accounted for 14% of the total, and both “COVID-19 patients seeking drugs” and “selling drugs” accounted for a relatively high percentage, indicating that there was a large demand for drugs within a short period of time after the liberalization of the pandemic policy. In the category of “selling drugs,” in addition to those who were trying to resell their own COVID-19 related medicines, there were also deceived buyers who posted information on their requests for help, reminding other netizens to be more careful in choose their sellers. The category of “seeking help to optimize management measures” accounted for 3%, which has been greatly reduced compared to the middle stage of the pandemic, indicating that the liberalization of the pandemic policy is a choice that conforms to the people’s wishes. “Other diseases patients seeking hospital treatment” accounted for only 1% in the later stage, which indicates that in the later stage, the hospital treatment of patients with other diseases is no longer affected by the COVID-19 pandemic, therefore the amount of this kind of posts has been reduced compared to the middle stage.

### 3.2 RQ2: comparison of the temporal distribution of help-seeking messages in different stages of the COVID-19 pandemic

**Figure 5:**
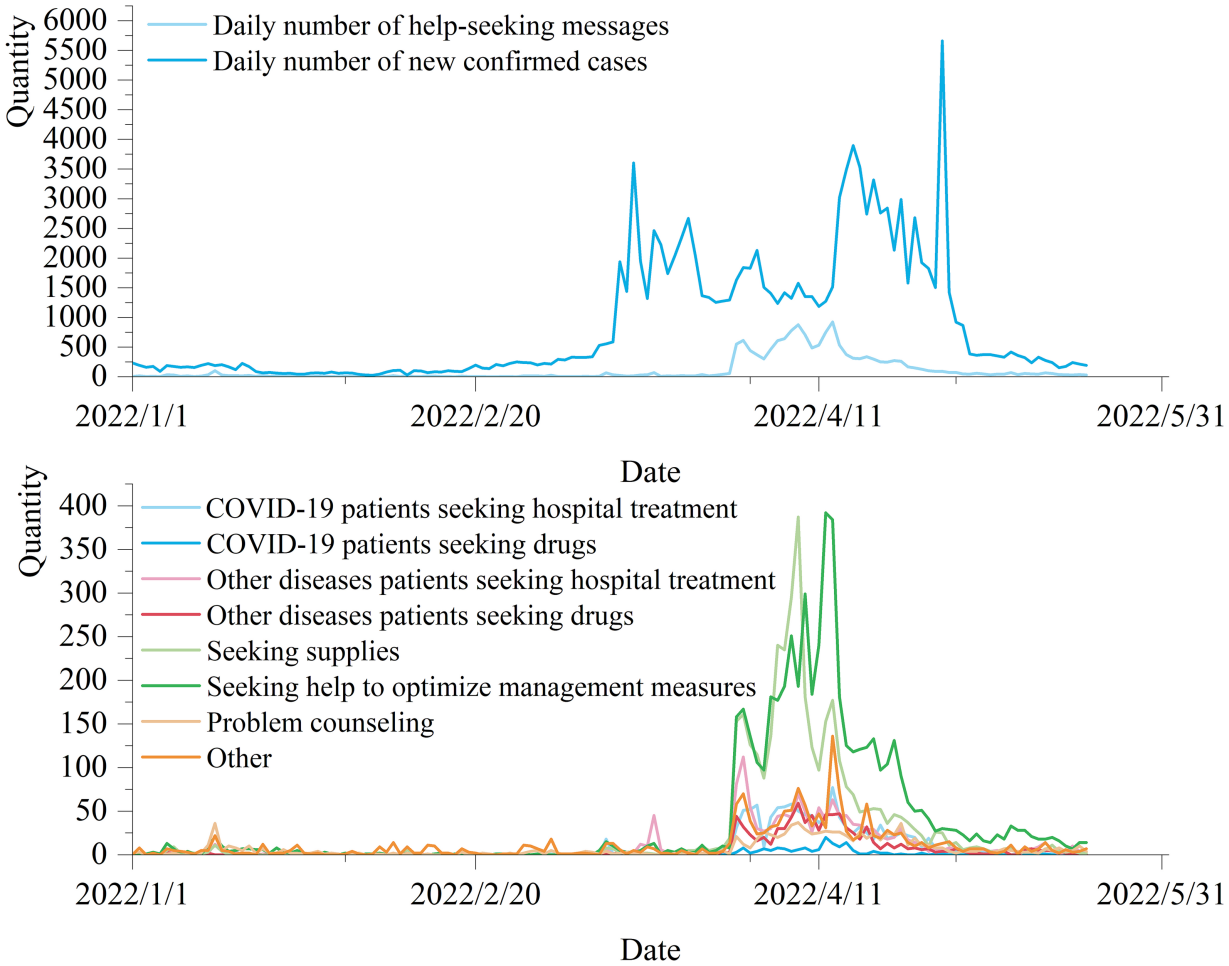
Time distribution of help-seeking information in the middle stage of the pandemic.

**Figure 6:**
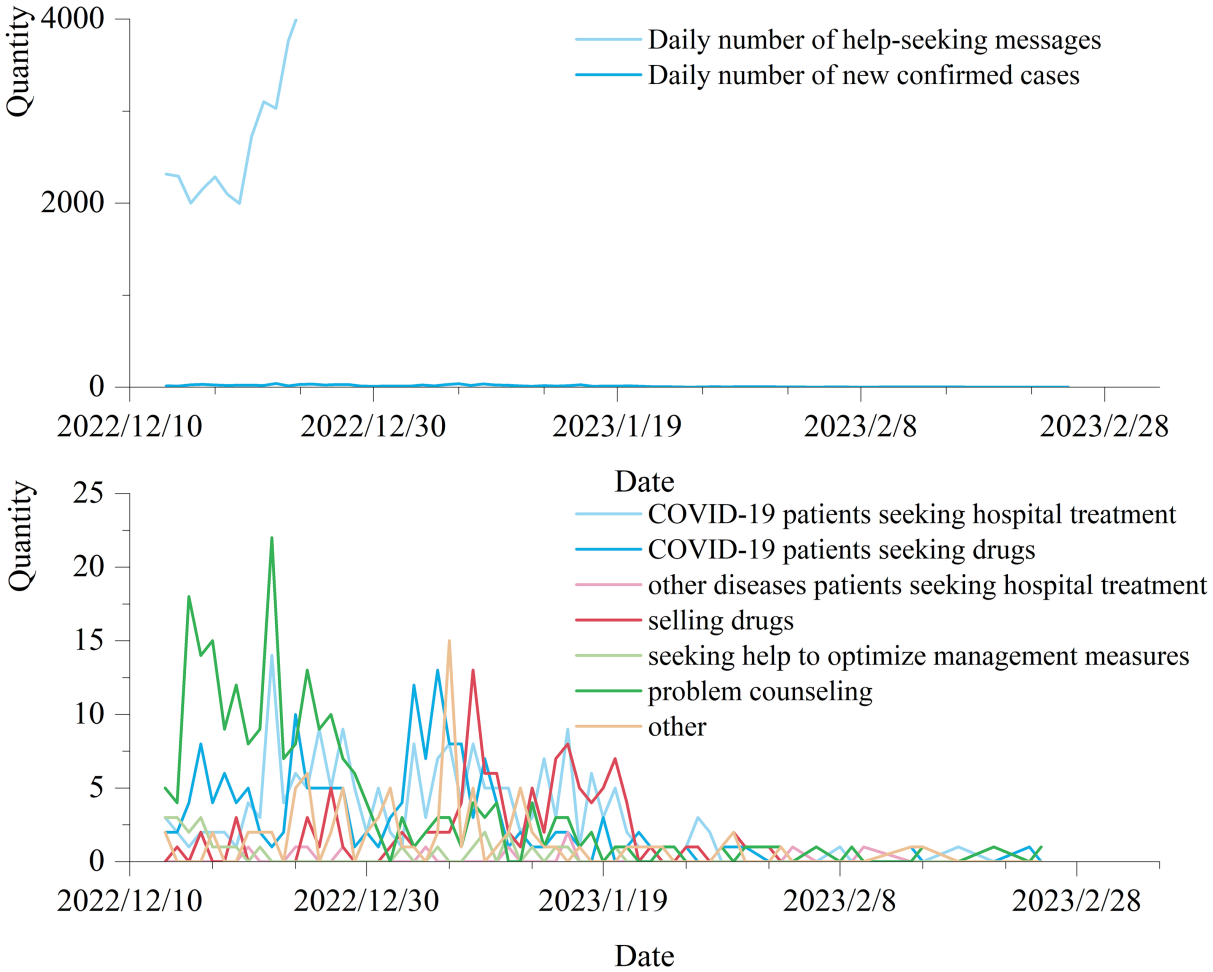
Time distribution of help-seeking information in the later stage of the pandemic.

Scholars’ study of Weibo help-seeking messages during the Wuhan pandemic found that the total number of daily help-seeking posts across China and Hubei were Granger causally related to the nation’s daily number of newly confirmed COVID-19 cases, and both had an 8-day time lag. In this paper, we statistically examine the changes in the number of daily help-seeking messages, and the number of new cases with date in the middle (Figure 5) and later stages (Figure 6) of the COVID-19 pandemic.

In the middle of the pandemic, (1) according to Figure 5, the number of daily new diagnoses of COVID-19 increased sharply on March 12, 2022, with the number exceeding 1,000, peaked at 5,659 on April 29, and plummeted to less than 1,000 on May 1; the number of daily help-seeking messages rose sharply on March 30, peaked on April 13, and then declined progressively. The situation is relatively better after May, and the daily help messages are maintained within 100. (2) The Time-Lag Cross-Correlation (TLCC) algorithm calculates the time lag between the number of new diagnose and the number of help-seeking messages (63, 64). The correlation formula is shown:


$$\left\{ \begin{array}{c} f_{\text{max}}\left(y_{1},y_{2}\right)=\left|f\left(y_{1},y_{2}+k\right)\right|\\ -N<k<N \end{array}\right.$$

From the formula, *f*(*y*_1_, *y*_2_) denotes the correlation between variables *y*_1_ and *y*_2_, and *N* denotes the total time, and the absolute value of the correlation reaches the maximum value *f*_max_(*y*_1_, *y*_2_) when *y*_2_ is lagged by order *k*. At this time, it can be said that there exists a time lag of time *k* between *y*_1_ and *y*_2_, in which the value of *k* is from −*N* to *N*.

**Figure 7:**
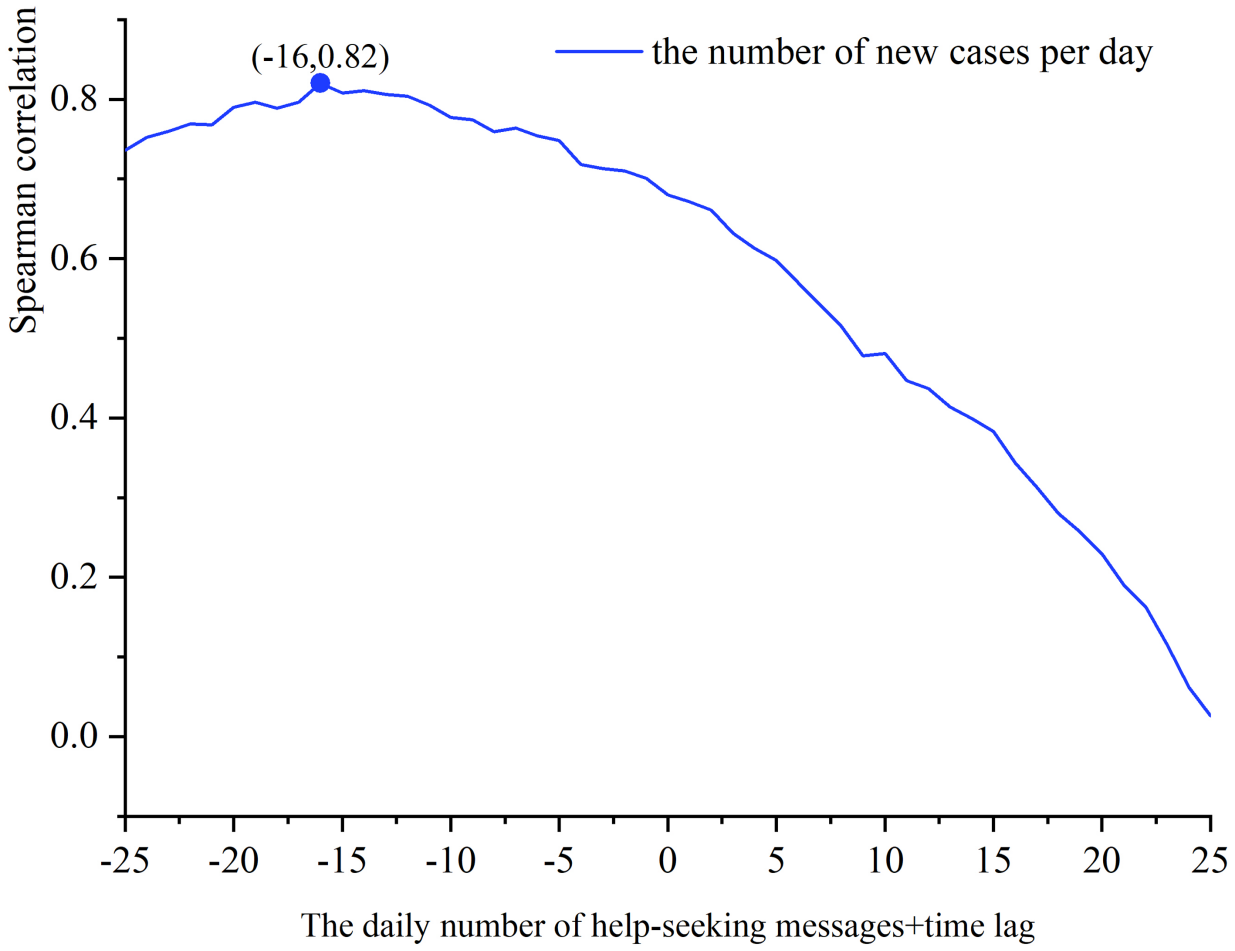
Spearman correlation and time lag.

Since neither the number of newly confirmed cases nor the number of daily help-seeking messages fit a normal distribution, we chose the Spearman correlation to calculate the correlation and time lag (65). According to the calculation of Spearman’s correlation, there is a significant correlation between the number of newly confirmed cases and the number of daily help-seeking messages with a value of 0.680. The time lag is further calculated and the result is shown in Figure 7, which shows that there is an 16-day time lag between the number of newly confirmed cases and the number of daily help-seeking messages in the middle stage of the pandemic, at which time the Spearman correlation coefficient is the largest, 0.820. More specifically, the number of newly confirmed cases lags the number of daily help-seeking messages by 16 days, and also shows that the number of newly confirmed cases can be predicted by the number of daily help-seeking messages. In the later stage of the pandemic, (1) according to Figure 6, the number of new cases increased exponentially after December 13, 2022, until China stopped counting this data on December 25, while the number of daily help-seeking messages remained within 40 per day, and then decreased to <10 per day after January 21, during which time there was no significant increase in the number of new cases. (2) There is no significant correlation or time lag between the number of new case and the daily number of help-seeking messages in this stage.

Combined with existing research on help-seeking messages, the number of newly confirmed cases and the number of daily help-seeking messages were significantly correlated in both the early and middle stages of the pandemic, with a time lag of 8 days in the early stage and 16 days in the middle stage. However, in the later stage of the pandemic, there was no significant correlation or time lag between the two.

### 3.3 RQ3: comparison of influencing factors of Weibo help-seeking message retweets in different stages of the COVID-19 pandemic

#### 3.3.1 Model comparison

Since the dependent variable is the number of retweets of help-seeking messages, which is a count variable, we initially chose Poisson regression model (66) and negative binomial regression model (66, 67). Immediately after that, considering the premise assumption of Poisson regression, that is, the mean of the explanatory variables is equal to the variance, which also restricts Poisson regression from applying to over-dispersed data. However, the variance of the data used in this paper is much more than the mean, so Poisson regression is ruled out, negative binomial regression model and its zero-inflated model are used in this paper.

Compared to Poisson regression, negative binomial regression relaxes the requirement that the variance is equal to the mean, in addition to this, it can deal with a moderate excess of 0. In the dataset of this paper, since the dependent variable of this model is the number of retweets of help-seeking messages, in the case of the medium-term help-seeking dataset, for example, 9,881 messages have a retweet number of 0. If the negative binomial regression is not enough to handle too many zeros, the zero-inflated negative binomial regression is usually considered (68). According to these two models, this paper compares their fitting effects based on three metrics, Log Likelihood, AIC and BIC, so as to select the optimal one (69, 70). The results of their indicators are shown in Table 5.

**Table 5:** Comparison of regression models.

	Log Likelihood		AIC		BIC	
	Middle period	Later period	Middle period	Later period	Middle period	Later period
Negative binomial regression	**−23977.62**	**−788.79**	**47991.23**	**1611.58**	**48128.55**	**1691.84**
Zero-inflated negative binomial regression	−24381.04	/	48800.08	/	48945.02	/

According to Table 5, in the middle stage of the pandemic, the absolute value of negative binomial regression is smaller than zero-inflated negative binomial regression in all three indicators, indicating that negative binomial regression fits better. In the later stage of the pandemic, the negative binomial regression fits well, while the zero-inflated negative binomial regression can not be fitted. Therefore, the negative binomial regression model is finally chosen as the model of this paper.

#### 3.3.2 Analysis of the regression results of the factors influencing the retweeting of Weibo help-seeking messages in different stages of the COVID-19 pandemic

Table 6 shows the descriptive statistics of all the data in the middle and later stages of the pandemic. Table 7 demonstrates the results of the negative binomial regression analysis of the factors influencing the number of retweets for all help-seeking messages. Both regressions were significant, with a middle period *r*^2^ of 0.190 and a later period *r*^2^ of 0.319.

**Table 6:** Descriptive statistics.

	Max		Mean		Median		Min		SD	
	Middle period	Later period	Middle period	Later period	Middle period	Later period	Middle period	Later period	Middle period	Later period
Retweets	70,959	8,206	29.987	19.419	0	0	0	0	664.835	299.402
*Content*										
Pictures and videos	1	1	0.311	0.305	0	0	0	0	0.463	0.461
Hashtags	1	1	0.451	0.384	0	0	0	0	0.498	0.487
@Others	1	1	0.229	0.184	0	0	0	0	0.420	0.388
Text length	3,337	3,999	201.960	221.649	144	132	6	7	204.104	356.071
Sentiment intensity	0	0	−6.040	−3.692	0	0	−3,861	−180	47.022	14.173
Content categories	8	9	5.152	4.881	5	7	1	1	1.748	3.133
*User*										
Followers	245,315,321	5,774,334	271347.505	65028.731	144	126.500	0	1	5551418.224	357,534
Verified	1	1	0.109	0.122	0	0	0	0	0.312	0.327
*Posting location*										
Super topic	1	1	0.603	0.505	1	1	0	0	0.489	0.500
*Speed*										
First retweeting time	1,000,000	1,000,000	692388.291	788228.488	1,000,000	1,000,000	0.167	1.200	461239.790	408475.352

**Table 7:** The results of the negative binomial regression.

	Coefficient		Standard error		*Z*-value		*p*-value	
	Middle period	Later period	Middle period	Later period	Middle period	Later period	Middle period	Later period
**Independent variable**								
*Content*								
Pictures and videos	1.611	1.044	0.052	0.278	31.028	3.751	0.000	0.000
Hashtags	−0.389	−0.466	0.243	0.240	−6.348	−1.600	0.000	0.110
@Others	1.174	−0.073	0.056	0.354	20.829	−0.493	0.000	0.622
Text length	0.001	0.001	0.000	0.000	8.786	2. 101	0.000	0.036
Sentiment intensity	0.000	0.013	0.001	0.006	0.198	2.073	0.843	0.038
*Content categories (based on COVID-19 patients seeking hospital treatment)*								
COVID-19 patients seeking drugs	−1.263	−0.044	0.247	0.295	−5.123	0.149	0.000	0.881
Other diseases patients seeking hospital treatment	−0.749		0.118		−6.329		0.000	
Other diseases patients seeking drugs	−0.164	0.674	0.094	0.647	−1.733	1.042	0.083	0.298
Seeking supplies	−0.684	−3.089	0.083	1.040	−8.229	−2.971,	0.000	0.003
Seeking help to optimize management measures	−1.452		0.093		−15.569		0.000	
Problem counseling	0.111	−1.780	0.122	0.329	0.909	−5.409	0.364	0.000
Other	−1.637	−1.733	0.112	0.565	−14.583	−3.070	0.000	0.002
Selling drugs		−2.357		0.513		−4.593		0.000
*User*								
Followers	−0.000	0.000	0.000	0.000	−0.647	4.875	0.517	0.000
Verified	0.323	0.628	0.073	0.357	4.405	1.758	0.000	0.079
*Posting location*								
Super Topic	0.111	1.532	0.056	0.310	1.979	4.940	0.048	0.000
*Speed*								
First retweeting time	−0.000	−0.000	0.000	0.000	−111.784	−23.232	0.000	0.000

For the middle stage of the COVID-19 pandemic, in the content dimension, a significant positive correlation was found between the number of retweets with having pictures or videos (coef = 1.611, *p* < 0.05), @others (coef = 1.174, *p* < 0.05), and the length of the text (coef = 0.001, *p* < 0.05). And there was a significant negative correlation between hashtags (coef = −0.389, *p* < 0.05) and the number of retweets. In content category dimension, using “COVID-19 patients seeking hospital treatment” as a base comparison, “COVID-19 patients seeking drugs” (coef = −1.263, *p* < 0.05), “other diseases patients seeking drugs” (coef = −0.749, *p* < 0.05), “seeking help to optimize management measures” (coef = −0.684, *p* < 0.05), “seeking supplies” (coef = −1.452, *p* < 0.05) and other (coef = −1.637, *p* < 0.05) are five categories getting less retweets, thus supporting hypotheses *H1*a, *H2*a, *H3*a and *H4*a, *H6*a. In the user dimension, a significant positive correlation was shown between user verification (coef = 0.323, *p* < 0.05) and the number of retweets, supporting hypothesis *H8*a. In the posting location dimension, help-seeking messages posted in a Super Topic (coef = 0.111, *p* < 0.05) received a higher number of retweets, supporting Hypothesis *H9*a. Finally, in the speed dimension, the quicker of the first retweet, the higher number of retweets (coef = −0.000, *p* < 0.05) a post could get, thus, proving Hypothesis *H10*a.

For the help-seeking messages in the later stage of the pandemic, in terms of content, having pictures or videos (coef = 1.044, *p* < 0.05) and text length (coef = 0.001, *p* < 0.05) had a significant positive effect on the number of retweets a post can get. The lower absolute value of sentiment intensity (coef = 0.013, *p* < 0.05) was associated with the higher number of retweets, supporting hypothesis *H5*b. In terms of content classification, using the category of “COVID-19 patients seeking hospital treatment” as a basis for comparison, “seeking help to optimize management measures” (coef = −3.089, *p* < 0.05), “problem counseling” (coef = −1.780, *p* < 0.05), and “selling drugs” (coef = −2.357, *p* < 0.05) and other (coef = − 1.733, *p* < 0.05), are the four categories getting fewer retweets, thus supporting hypotheses *H1*b, *H4*b, and *H6*b. In the user dimension, the higher the number of followers (coef = 0.000, *p* < 0.05), the higher the number of retweets obtained, and hypothesis *H7*b was supported. Next, in the posting location dimension, there is a significant positive correlation between Super Topic (coef = 1.532, *p* < 0.05) and the number of retweets, supporting hypothesis *H9*b. Finally, in the speed dimension, the quicker to get the first retweet (coef = −0.000, *p* < 0.05), the more retweets will be obtained, supporting hypothesis *H10*b.

## 4 Discussion

This paper explores the changes in content categories, time distribution, and retweeting influencing factors of help-seeking messages on Weibo during three different time periods of the COVID-19 pandemic in China, complementing existing studies on help-seeking messages during the latter two periods of the pandemic and providing a multi-period and multi-dimensional comparison. The results shows that help-seeking messages in the three dimensions of content categories, time distribution, and retweeting influencing factors exhibit different characteristics as the pandemic develops.

In the content categories dimension, the focuses of people’s help-seeking posts varied in different periods. In the middle stage of the COVID-19 pandemic, the number of requests for hospital treatment and drugs for other diseases exceeded the number of requests for medical resources for COVID-19 patients. In the later stage, there were far more COVID-19 patients seeking hospital treatment and drugs than other diseases, and the proportion of COVID-19 patients seeking drugs increased significantly in the later stage, while the categories of patients seeking drugs and supplies for other diseases had disappeared in the later stage. In addition, the percentage of problem counseling category has increased and the posts of selling drugs has appeared in the later stage.

In the time distribution dimension, the temporal distribution of Weibo help-seeking messages in the middle and later stages is plotted and compared with the daily newly confirmed cases. The correlation and time lag between the two are calculated by TCLL. It is found that there is a significant correlation between the daily help-seeking messages and the daily newly confirmed cases in the middle stage of the pandemic, and there is a 16-day time lag. In contrast, there is no correlation between daily help-seeking messages and new cases per day in the later stage, and the overall number of help-seeking messages in the later stage was significantly lower than that in the other stages of the pandemic.

In the retweet influencing factors dimension, combined with existing research on help-seeking messages in the early period, we find that having pictures or videos and text length significantly positively affect the number of retweets in each period. Hashtags did not significantly affect retweets in early and later periods, but they significantly negatively affected retweets in the middle period. @Others only had a significant positive effect on the number of retweets in the later period. Emotional intensity had no significant effect on the number of retweets in the early and middle stages of the pandemic, and the lower the absolute value of emotional intensity, the higher the number of retweets in the later period. The number of followers significantly affected retweets in the early and later periods, but not in the middle period. User verification had a significant effect on getting retweets in the early and middle stages, but it’s not the case in the later stage. Super-Topic did not significantly affect retweets in the preliminary study, but did have affect in the middle and later periods, probably due to the management and the increased influence of super-topic during the Changchun and Shanghai pandemics. Although there was no accelerating speed-related factor in the preliminary study, our study on the middle and later stage shows that the time of the first retweeting can significantly affect the number of retweets of help-seeking messages.

Previous studies on help-seeking messages have mostly focused on the early stage of the COVID-19 pandemic, while the middle and later stages have not been sufficiently researched since the pandemic lasted for 3 years. To address the above issues, this paper fully investigates the Weibo help-seeking messages in the middle and latter stages from the dimensions of content categories, time distribution, and retweet influencing factors, which fills the gap in the existing studies. More importantly, existing research lacks a long-term, multifaceted study of online help-seeking messages during the pandemic period, while this paper compares the three periods of the pandemic in multiple dimensions, covering the beginning of the pandemic as well as the release of the pandemic liberalization policy, which makes up for the shortcomings of the existing research in this field.

In addition, the findings of this paper are also significant. The study found that the focus of help-seeking information varies in different periods. There is no correlation between help-seeking information and newly confirmed daily cases in the later stage of the COVID-19 pandemic, but there is a correlation and time lag between the two in the other periods. In terms of the factors influencing retweets, the factors significantly affecting retweets varied across time, with pictures or videos, and text length significantly affecting retweets in each period. These findings fully demonstrate the evolution of online help-seeking behavior during the COVID-19 pandemic. Many scholars have already focused on exploring and proving the changes in policies, perceptions, and behaviors during the pandemic, and this paper undoubtedly adds another strong empirical proof and makes an important academic contribution.

Since the beginning of the pandemic, China has adopted strict control policies. Showing great respect as a responsible big country, China has always put life first and has been very effective in reducing the number of infections and deaths (71). The World Health Organization (WHO) has praised China’s rapid and robust prevention and control initiatives and recommended that other countries follow suit (72).

However, this paper finds that there is room for improvement in China’s policies when examining online help-seeking messages. The study of the middle stage of the pandemic found that the problem of blocked access to medical care and medication for patients with other diseases that occurred during the closure and control should not be ignored, and that the strict isolation standards caused considerable distress to patients with other diseases, which reminded the government of the need to provide these patients with specialized policy assistance in the future when they encountered the same situation, so as to avoid delays in their treatment.

Furthermore, insufficient supply of resources is also one of the problems manifested in the early, middle and later periods. Resources include medical resources such as hospital beds and medicines, as well as daily supplies such as vegetables and daily necessities. Insufficient supply of medical resources is unavoidable in the face of such serious public health emergencies, but the government still needs to accumulate experience so that it can make a better response to similar situations in the future. Community distribution of supplies is especially critical when transportation is blocked during closures, but there are still problems with the stockpiling and dispatching of supplies, resulting in help-seekers having to post their requests for help online, so the government’s supply and dispatching of supplies needs to be further matured and improved in the face of public health emergencies.

In addition to this, the government should increase its attention to online help-seeking messages. In the early and middle stages, the number of help-seeking messages is closely related to the number of new confirmed cases. Governments need to focus on the commonalities in help-seekers’ problems in order to achieve better management. Social media should also take responsibility and cooperate with the government to contribute to solve the real problems of help-seekers.

The research on the retweeting factors of help-seeking messages in this paper is not only to prove the changes of help-seeking messages in different periods, but also to find out the stabilizing factors among the ones affecting the retweeting of help-seeking messages. Although the other factors show less stable performance, pictures and videos, and the length of the text significantly affect the amounts of retweets in each period. This also provides suggestions for help-seekers when posting help messages: having pictures and videos, and more text length will result in more retweets, thus increasing the chances of being helped.

### 4.1 Limitations

The impact of policies on China’s pandemic is also significant, but unfortunately, this paper does not cover the empirical study of the impact of policies and help-seeking messages. In terms of help-seeking messages retweeting factors, in addition to the negative binomial regression as a method, the use of neural networks can also be considered. For future research, scholars can dig deeper into the psychological as well as social factors that led to the changes in these help-seeking messages, and they can also verify the impact of policies on help-seeking messages. In addition, scholars can explore other factors that evolved during the pandemic.

## 5 Conclusion

This paper examines the changes of Weibo help-seeking messages during different periods of the COVID-19 pandemic in three dimensions: content categories, time distribution, and retweeting influencing factors. In the content categories dimension, the content focuses are different in the early, middle, and later periods, and all of them reflect the social needs and social problems in different periods. In the time distribution dimension, there are significant correlations and time lags between new daily help-seeking messages and new confirmed cases in the early and middle stages of the pandemic, which are 8 and 16 days, respectively, but there is no correlation in the late period. In the retweeting influencing factors dimension, pictures or videos and the length of the text significantly and positively influence the amount of retweets in all periods, although some of the factors behave erratically. The conclusions drawn in this paper in content categories can help policy makers to have a clearer understanding of the needs of the society and help to fill the gaps in the policies, as well as to gain experience for possible public health emergencies in the future. At the same time, the conclusions in this paper can provide advice to help-seekers, which can help them get more retweets when they post help-seeking messages. Finally, this paper presents another unique perspective of the COVID-19 pandemic in China at different times to help readers understand the pandemic more deeply.

## Data availability statement

The original contributions presented in the study are included in the article/supplementary material, further inquiries can be directed to the corresponding author.

## Author contributions

JJ: Conceptualization, Funding acquisition, Methodology, Writing – original draft, Writing – review & editing. CY: Conceptualization, Data curation, Methodology, Writing – original draft, Writing – review & editing. XS: Data curation, Writing – review & editing.
